# Nursing-Led Interventions for Preventing Falls in Hospitalized Patients: A Systematic Literature Review

**DOI:** 10.3390/nursrep16070232

**Published:** 2026-07-03

**Authors:** José Moreira, Patrícia Fialho, Sílvia Alexandrino, Marisa Mendes, Lina Granadeiro, Helga Martins, Susana Miguel

**Affiliations:** 1 Escola Superior de Enfermagem São João de Deus, Universidade de Évora, 7000-811 Évora, Portugal; silviacarmo@hotmail.com; 2 Comprehensive Health Research Center (CHRC), Universidade de Évora, 7000-811 Évora, Portugal; 3 Unidade Local de Saúde do Alentejo Central, 7000-811 Évora, Portugal; patricia.ip.fialho@gmail.com (P.F.); lina_granadeiro@hotmail.com (L.G.); 4 Centre for Interdisciplinary Research in Health, Faculty of Health Sciences and Nursing, School of Nursing, Universidade Católica Portuguesa, 7000-811 Lisbon, Portugal; hemartins@ucp.pt (H.M.); ssmiguel@ucp.pt (S.M.)

**Keywords:** accidental falls, health education, incidence, hospitals, inpatients, nursing care, patient safety, risk assessment, systematic review

## Abstract

**Background:** In-hospital falls are common adverse events associated with injuries, functional decline, prolonged length of stay, and increased healthcare costs, which require effective and sustained nursing interventions. **Objective:** To identify, through a Systematic Literature Review, which nursing care interventions are effective in reducing the incidence/rate of falls among inpatients in hospital settings. **Methods:** A systematic literature review was conducted using the JBI methodology. The review was guided by the PICO framework (P: inpatients; I: nursing care interventions; C: usual care; O: incidence of accidental falls). A comprehensive search was performed in the MEDLINE, CINAHL, and Scopus databases. Studies were included if they evaluated nursing-led or nursing-related interventions aimed at fall prevention and reported fall-related results. Eligible study designs included randomized controlled trials, quasi-experimental studies, observational studies, and quality improvement initiatives. Study selection, data extraction, and critical appraisal were conducted according to JBI recommendations. **Results:** Six studies were included (quasi-experimental, cohort, prospective/observational, and quality improvement projects). Two main themes emerged: (1) structured multifactorial and educational interventions and (2) technology-based interventions. Multifactorial approaches that combine risk assessment, education, communication, and environmental measures have been shown to improve adherence and reduce falls. Technology-based interventions, especially video monitoring, showed the most consistent reductions in fall rates, including fewer nighttime falls and decreased need for one-to-one observation. The included studies were methodologically heterogeneous in design, clinical setting, and outcome definitions, which precluded statistical pooling and warrants caution in the interpretation of the findings. **Conclusions:** Structured, standardized, multifactorial, and nursing-led approaches can contribute to reducing inpatient falls. However, more robust and comparable studies are required to consolidate practice-relevant recommendations.

## 1. Introduction

Falls among hospitalized patients represent a frequent and clinically significant adverse event in the hospital setting, being associated with an increased risk of injury, prolonged hospital stays, functional decline, and higher healthcare costs [1,2]. Despite the existence of risk assessment strategies and preventive measures, the incidence of falls remains considerable, particularly among older patients and those with high clinical complexity [1]. This challenge reinforces the need for more effective and consistent interventions in inpatient care settings.

Fall prevention is a structured and multidimensional process that requires the integration of combined interventions involving risk assessment/stratification, patient safety education, environmental adaptation, process evaluation, and reinforcement of surveillance and monitoring practices [1,3]. Within this context, nurses play a central role, owing to their continuous proximity to patients, ability to identify risk factors at an early stage, and capacity to implement individualized and patient-centered interventions. Evidence from quality improvement initiatives and multifactorial intervention studies conducted in inpatient settings suggests that such approaches can effectively improve clinical indicators and reduce the incidence of falls, including those resulting in injury, particularly when they are embedded within standardized protocols and supported by ongoing monitoring and evaluation processes [4,5,6,7].

In recent years, the literature has shown that nursing-led operationalized interventions, with the involvement of the multidisciplinary team, can generate measurable improvements in patients’ outcomes. Continuous quality improvement initiatives and team-based intervention strategies have been described as viable and effective approaches, particularly when they incorporate interprofessional coordination and standardization of care procedures [7]. Simultaneously, nursing-led interventions with a temporal or organizational focus, such as nurse-led models and multicenter initiatives, can address the specific dynamics of healthcare services and periods of increased risk [8,9].

Clear leadership, supported by organizational management and the ongoing training of multidisciplinary teams, is widely recognized as essential for the practical and sustainable implementation of fall prevention measures. These elements facilitate their functional integration into routine clinical practice, helping to ensure that prevention strategies are applied consistently and effectively rather than reduced to purely bureaucratic or procedural tasks [10,11].

Educational interventions are also highly relevant, as they promote safety literacy and adherence to preventive measures [4,7]. It is equally important to highlight the role of patient education and the training of healthcare professionals [4,7]. Innovative educational approaches, such as using video and simulation, have also been studied, showing reductions in fall rates and/or in fear of falling among hospitalized older adults [12].

Structured communication strategies, including methodologies such as teach-back, have been applied in rehabilitation and inpatient settings with a measurable impact on fall reduction, as they strengthen patients’ understanding and engagement in safety measures [13]. However, their active participation may be limited by a lack of risk awareness or fear of bothering healthcare professionals, which underscores the need for individualized, empathetic communication tailored to the patient’s profile, thereby promoting conscious engagement in prevention strategies [10,14].

Among fall prevention measures, the relevance of environmental and safety modifications has been highlighted, including improved lighting, the use of motion sensors, and systematic removal of obstacles, particularly during periods of greater vulnerability such as the night shift [11,14]. Additionally, technology-based and environmental safety interventions have gained prominence, particularly video surveillance/monitoring during periods of increased risk, including the night shift, and continuous monitoring programs in settings characterized by high complexity and frequent patient mobilization have been increasingly implemented [15,16]. Furthermore, measures related to footwear safety and adherence (e.g., non-slip socks versus adequate footwear) have also been explored as components of a comprehensive prevention approach for hospitalized patients [17].

Despite the available evidence, heterogeneity in the implementation of interventions and variability across contexts, populations, and resources persist, which may affect the effectiveness and sustainability of programs. Although recent systematic reviews have addressed fall prevention in hospitals, they have predominantly pooled heterogeneous multidisciplinary interventions and have rarely isolated the specific contribution of nursing-led interventions, nor focused on the most recent evidence published after the 2022 clinical practice guideline. This represents a knowledge gap that the present review aims to address by synthesizing recent evidence with an explicit focus on nursing-led and nursing-related interventions. Therefore, the systematization of the literature is essential to identify which nursing interventions are most effective in reducing the incidence/rate of falls among hospitalized patients, thereby supporting evidence-based practice and promoting patient safety.

This systematic literature review aimed to identify and analyze nursing interventions that are effective in reducing the incidence/rate of falls among hospitalized patients. Considering this, we formulated the following PICO question: Which nursing interventions are effective in reducing the incidence/rate of falls among hospitalized patients?

## 2. Materials and Methods

This systematic review followed the JBI methodology for systematic reviews [18] and was reported using the Preferred Reporting Items for Systematic Reviews and Meta-analyses (PRISMA) guidelines [19]. The protocol is registered in the PROSPERO database under the registration number CRD420261424557 and is available at: https://www.crd.york.ac.uk/PROSPERO/view/CRD420261424557 (accessed on 15 June 2026).

### 2.1. Eligibility Criteria

Study selection was based on the PICO framework (Population, Intervention, Comparison, Outcome), as recommended by the JBI methodology for systematic reviews assessing the effectiveness of interventions [18]. The comparator (C) was usual care, that is, the standard fall-prevention practices in place at each setting prior to, or in the absence of, the evaluated nursing intervention (including pre-implementation periods, concurrent control wards, or historical reference periods). Studies without an identifiable comparison condition were not eligible. The full PICO framework is also presented in Supplementary Table S1.

#### 2.1.1. Type of Participants

Studies involving adults aged ≥ 18 years admitted to a hospital, regardless of the clinical department or specialty, were eligible for inclusion. Studies were excluded if they focused exclusively on outpatient populations, long-term care facilities without a hospital component, or paediatric populations (aged < 18 years).

#### 2.1.2. Intervention

The phenomenon of interest was nursing interventions implemented in hospital settings that aimed to prevent or reduce the incidence of falls. Studies were considered eligible if they described or evaluated nursing interventions, whether delivered as standalone measures or integrated within multidisciplinary programs, and reported fall incidence or fall rate among hospitalized patients as an outcome. Studies were excluded if no identifiable nursing intervention was present, if they lacked relevance to the research object, or if the methodology was ambiguous or insufficiently reported.

Studies conducted in hospital settings, specifically in inpatient units of acute care hospitals, were considered eligible regardless of country or healthcare system. The search was limited to the period from January 2021 to December 2025. This timeframe was defined for two complementary reasons: (1) to ensure the currency of the evidence retrieved; and (2) to incorporate evidence published after the release of the clinical practice guidelines on fall prevention in hospitals and nursing homes by Schoberer et al. [14] in 2022. The primary aim of this temporal restriction was to synthesize the evidence generated after the publication of these clinical practice guidelines. We acknowledge that limiting the search to 2021–2025 may have excluded relevant earlier studies; this restriction is therefore explicitly recognized as a limitation of the review (see Section 4).

#### 2.1.3. Outcome

The primary outcome was the incidence of accidental falls among hospitalized adults, including measures such as fall incidence, fall rate per 1000 patient-days, and the proportion of patients who experienced falls. For the purposes of this review, the fall rate was defined as the number of falls divided by the number of patient-days (or bed-days) multiplied by 1000. We note that the definitions and denominators used to express fall outcomes (e.g., number of falls versus number of patients who fell, and whether recurrent falls in the same patient were counted separately) varied across the included studies; these definitions were extracted as reported by each study and were not harmonized, which is acknowledged as a source of heterogeneity (see Section 4). Secondary outcomes included changes in validated fall-risk assessment scores, adherence to fall-prevention protocols, and process indicators related to the implementation of nursing interventions.

#### 2.1.4. Type of Studies

Studies with methodological designs enabling the assessment of the effectiveness of nursing interventions in fall prevention were eligible. These included experimental studies (randomized and non-randomized controlled trials), quasi-experimental studies, observational studies (cohort, case–control, and cross-sectional), and quality improvement studies. Systematic reviews, meta-analyses, editorials, letters to the editor, case reports, opinion pieces, and narrative review articles were excluded. Additionally, studies published before 2021, articles without full-text availability, and articles written in languages other than English or Portuguese were also excluded. Studies that did not provide sufficient fall-related outcome data were also excluded.

### 2.2. Search Strategy

The search strategy was built around the PICO framework, with the following components: population—comprising adults aged 18 years or older admitted to a hospital setting; intervention—nursing interventions for fall prevention; comparison—usual care; outcome—incidence of accidental falls. The search was conducted through the EBSCO electronic platform for publications between January 2021 and December 2025, across the following databases: CINAHL (Cumulative Index to Nursing and Allied Health Literature) full-text, MEDLINE, and Scopus. The search terms used, along with the Boolean operators and filters applied to each database, are listed in Table 1. Filters for full-text availability, language (English and Portuguese), and publication date (January 2021 to December 2025) were applied. The search was conducted from January 2021 to December 2025. Controlled vocabulary (exact subject headings/MeSH-equivalent terms, indicated as [a] in Table 1) was combined with free-text title/abstract terms ([b]) and title/abstract/keyword terms in Scopus ([c]) using the Boolean operators OR (within each PICO component) and AND (between components), with truncation (*) applied to capture—word variants. The complete, database-specific search strings are provided in Supplementary Table S2.

### 2.3. Study Selection

Randomized, quasi-experimental, observational (prospective and retrospective) studies and quality-improvement projects were included, provided they evaluated nursing interventions or nursing care implemented in hospitalized patients and reported fall incidence/rate as an outcome. The search results were imported into the Rayyan^®^ platform [20], where duplicate records were identified and removed automatically. Subsequently, two independent reviewers (PF and SA) blindly applied the predefined eligibility criteria. Study relevance was first determined through title and abstract screening. The full texts of potentially eligible articles were independently assessed by both reviewers, as mentioned above. The level of agreement between the two reviewers at the full-text selection stage was quantified using Cohen’s kappa coefficient (κ = 0.35); all discrepancies were subsequently resolved by consensus, with the third reviewer (JM) consulted when required.

### 2.4. Data Extraction

Data extraction was conducted by a primary reviewer (PF) using a structured instrument developed in accordance with the review objective to collect and systematize essential information from the included studies. The extracted data were summarized in both tabular and narrative formats and included the following variables: author and year of publication, country, study design and sample, population and setting, period and duration, nursing interventions, comparator, measures/instruments/outcomes, follow-up period, results, and JBI methodological quality. The extraction was subsequently reviewed by a second independent reviewer (SA) to ensure consistency and reliability of the findings. Given the methodological and clinical heterogeneity of the included studies (differences in study design, interventions, settings, and outcome definitions), a meta-analysis was not appropriate. Therefore, the results were analyzed and synthesized using a narrative and thematic synthesis approach: the extracted data were grouped by type of intervention, and recurrent patterns were organized into the two themes presented in Section 4, with the magnitude and direction of the reported effects summarized for each study.

### 2.5. Quality Assessment

The methodological quality of each study was assessed individually in accordance with the JBI recommendations, using the appropriate critical appraisal tools for each study design. The overall appraisal was summarized in both narrative and tabular formats, allowing the identification of methodological quality and risk of bias associated with the included research (RoBANS—Risk of Bias Assessment tool for Non-randomized Studies). The total score was calculated as the proportion of criteria met within each checklist. To clarify, two complementary instruments were used for distinct purposes. The design-specific JBI critical appraisal checklists were used to derive the overall methodological quality score for each study, applying the checklist corresponding to each design (quasi-experimental, cohort, prospective/observational, and analytical cross-sectional/quality-improvement studies). As all included studies were non-randomized, the RoBANS tool was additionally used to assess the domain-specific risk of bias reported in Table 2. Percentage-based categorization (moderate, high, excellent) was adopted, following previous JBI-based reviews, to allow a transparent and comparable summary of the proportion of appraisal criteria met across heterogeneous study designs, and was used descriptively rather than as a threshold for inclusion or exclusion. A score between 70 and 79% was considered moderate quality, between 80 and 90% indicated high quality and scores above 90% were classified as excellent [21,22]. No study was excluded on the basis of its risk-of-bias rating. Domains judged to be at high or unclear risk of bias (such as blinding of outcome assessment, incomplete outcome data, or confounding) and outcomes that were not reported were not imputed or pooled; instead, they were flagged in Table 2 and taken into account qualitatively when interpreting the strength and consistency of the evidence, with the corresponding findings interpreted with additional caution in the Discussion.

### 2.6. Ethical Procedures

Although this systematic review did not require formal ethical approval, ethical rigor was ensured by strictly adhering to established research methodologies. Particular attention was paid to ensuring the transparency and accuracy of this systematic review, thereby upholding the integrity of the research process.

## 3. Results

### 3.1. Identified Studies and Quality Appraisal

**Figure 1:**
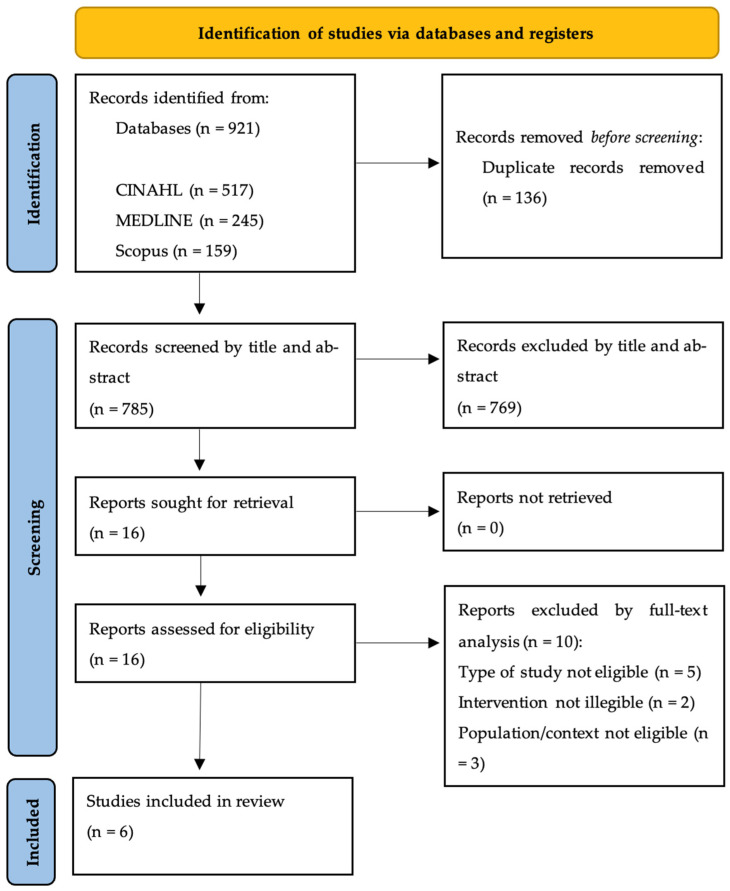
Flow Diagram of article selection (adapted from guidelines for the development of systematic reviews) [19].

Searches of electronic databases identified 921 records (Figure 1). After removing 136 duplicates, 785 records were screened. Following title and abstract screening, 769 records were excluded, leaving 16 reports assessed for full-text eligibility. After full-text assessment, six studies that met the inclusion criteria were included, with descriptions/reports of nursing interventions performed on hospitalized patients in a hospital setting, presenting the incidence or rate of falls as an outcome. Figure 1 shows the PRISMA 2020 flowchart, which summarizes the process of identifying, selecting, and including studies.

The methodological quality of the studies included ranged from moderate to high, with JBI appraisal scores ranging between 73.7% and 88.9%. Specifically, two studies were rated as moderate quality Rodríguez et al. (8/11, 73.7%) [26] and the remaining studies as high quality: Yost & Baur, Woltsche et al., and Kalafus et al. (9/11; 81.8%) [15,23,25], and Akhiwu et al. [24] and Miles et al. (8/9; 88.9%) [27].

The risk of bias assessment indicated that the overall methodological quality of the studies included in the systematic literature review was generally acceptable, with most articles showing a low risk of bias across several domains (Table 2). In particular, some studies were rated as having a low risk for intervention measurement and selective outcome reporting, suggesting consistency in how interventions were assessed and results were reported. However, some concerns were identified in other domains, especially regarding blinding of outcome assessment, incomplete outcome data, and confounding variables, where several studies showed either high or unclear risk of bias.

### 3.2. Characteristics of Studies

The six studies included in this review (Table 3) were published between 2021 and 2025. The studies were predominantly conducted in the United States (n = 3; 50%) [23,24,25], Australia (n = 2; 33.3%) [15,27], and Spain (n = 1; 16.7%) [26].

The included studies encompassed a range of methodological designs, including continuous quality improvement projects [23,24], a cohort study [15], prospective observational studies [25,26], and a quasi-experimental study [27], all conducted in hospital settings. All studies evaluated nursing interventions, either delivered as standalone strategies or integrated into multidisciplinary programs, and reported fall incidence or fall rates as primary outcomes. The interventions were implemented across a variety of clinical settings, including medical-surgical units [23,24], rehabilitation settings [27], acute care units [25], geriatric units [15,27], and geriatrics and internal medicine units [26].

Across the six included studies, two major categories of nursing interventions emerged: (1) structured multifactorial and educational interventions and (2) technology-based interventions.

#### 3.2.1. Structured Multifactorial and Educational Interventions

Four studies [23,24,26,27] evaluated interventions centred on the organisation of care processes, nurse-led education, and structured communication strategies. These approaches share a common emphasis on standardizing nursing practice, enhancing knowledge of both patients and staff, and promoting adherence to fall prevention measures [23]. The use of bundles was one of the strategies implemented, which incorporated nursing staff education and training, bed and chair alarms, and yellow identification wristbands [23]. Akhiwu et al. implemented structured education for patients, families, and healthcare professionals [24], whereas Miles et al. [27] developed a communication-focused intervention targeting the entire multidisciplinary team. In contrast, Rodríguez et al. [26] implemented an intervention specifically centered on appropriate footwear.

#### 3.2.2. Technology-Based Interventions

Two studies employed technology-based monitoring interventions [15,25]. Woltsche et al. implemented portable nocturnal video monitoring in hospitalized adult patients at high risk of falls [15], while Kalafus et al. [25] used a continuous video monitoring system, complemented by activation protocols and nursing coordination procedures to ensure rapid response. Together, these interventions aim to enhance real-time surveillance, support timely clinical decision-making, and reduce fall events through technology-assisted oversight. Across the six studies summarized in Table 3, nursing interventions produced diverse but generally favorable effects on fall-related outcomes. Yost and Baur [23] reported an 8% reduction in fall rates following the implementation of a standardized assessment tool, bundled precautions, and patient/family education. Technology-supported monitoring showed the most substantial impact: Woltsche et al. [15] achieved a significant reduction in nighttime falls (from 4.54 to 2.26 per 1000 bed-days; *p* = 0.003), while Kalafus et al. [25] observed decreases in monthly falls (17.2 to 12.9; *p* = 0.02) and a marked reduction in 1:1 sitter hours after introducing continuous video monitoring. Educational and communication-focused interventions yielded mixed results: Akhiwu et al. [24] improved adherence and documentation but did not reduce fall incidence, whereas Miles et al. [27] found that a teach-back communication strategy reduced falls in specific subgroups (low-risk patients and those without cognitive impairment). Interventions used by Rodríguez et al. [26] showed that all recorded falls occurred among patients wearing their own footwear, while none occurred in the non-slip sock group.

## 4. Discussion

Fall prevention in nursing is a highly relevant and evolving field that has undergone significant development in recent years. This systematic literature review provides a strong scientific foundation for understanding the effectiveness of fall prevention interventions, particularly regarding the extensive body of existing evidence on this topic. From a comparative perspective with previous systematic reviews, there is a clear trend towards the increasing implementation of nursing interventions that are specifically oriented toward the digital dimension [29]. These findings are consistent with current healthcare trends, which reflect the growing integration of technology into clinical practice [30].

Multifactorial and structured care interventions, such as those described by Yost et al. [23], demonstrated modest but meaningful reductions in fall incidence (8%). This is consistent with other studies; for instance, Dykes et al. [31] reported that bundled interventions combining risk assessment tools, environmental modifications, and patient education achieved a 15% reduction in falls and a 34% reduction in injuries. These findings reinforce the notion that standardisation of nursing processes and adherence to evidence-based protocols are critical determinants of success.

However, similar to Akhiwu et al. [24], several studies have shown that improvements in staff knowledge, documentation, and protocol adherence do not always translate into statistically significant reductions in falls, suggesting a gap between process outcomes and clinical outcomes [32] In addition, evidence indicates that accurately translating these programs into routine practice is challenging, and the effectiveness of such interventions is highly context-dependent [33]. Consequently, improvements in processes such as staff training, adherence to protocols, or documentation do not necessarily result in measurable reductions in fall incidence [33]. A key factor influencing the effectiveness of fall prevention interventions is the level of adherence when operationalized in practice. For instance, Spoon et al. [34] reported that median adherence to prescribed protocols was only 65%, which may partly explain the modest impact on fall incidence. On the other hand, the clinical environment plays a critical role in determining intervention outcomes. Evidence from Wyss-Hänecke et al. [33] demonstrates that similar interventions can produce markedly different effects depending on contextual factors such as ward characteristics, patient dependency levels, and organisational processes.

Focusing on the fundamentals, starting with the simplest interventions. The findings of Rodríguez et al. [26], who demonstrated the protective effect of non-slip socks, are consistent with recent findings that reported reductions in fall risk associated with appropriate footwear interventions. A comprehensive review by Kim and Hegazy [35] found that certain footwear design features (fit, fixation, slip resistance) can influence balance and may help reduce fall risk among older adults. However, the review shows that caution about footwear is only one component of fall risk and must be considered with other factors such as gait stability and balance support [35].

Technology-supported monitoring showed among the most consistent reductions in fall rates in the included studies; however, this evidence derives mainly from observational and quasi-experimental designs, without randomized controlled trials, so these findings should be interpreted with caution and not as evidence of the definitive superiority of technological over multifactorial interventions [25,28]. For instance, Woltsche et al. [15] reported a significant reduction in nighttime falls. These findings are consistent with those of similar studies. A similar study by Wright and Singh [36] showed that vision-based monitoring systems reduced nighttime falls by 48%, while also decreasing fall-related injuries and hospital resource utilization.

The existence of studies focusing specifically on the use of vision-based monitoring during nighttime is particularly relevant, as evidence suggests that falls are more likely to occur during this period [29,37]. This increased risk is associated with environmental and organizational factors, including reduced staffing levels, environmental hazards, patient disorientation, toileting needs, and increased mobility [38]. In this context, the implementation of technological monitoring systems represents an effective strategy for mitigating these risks, especially during night shifts when supervision is more limited.

However, some limitations related to the implementation of these technologies must be considered. In particular, the high initial costs of acquiring and maintaining such equipment can represent a significant barrier for healthcare institutions [39]. Nevertheless, it is important to consider a broader economic perspective. Patient falls are associated with increased healthcare costs, including prolonged hospital stays, additional diagnostic tests, and, in some cases, surgical interventions [40].

Therefore, despite the initial investment required, the long-term benefits of implementing technological monitoring systems are likely to outweigh the costs, as mentioned in the study by Kalafus et al., which showed a $3.2 million cost savings due to fewer falls and reduced sitter use [25]. These systems not only improve patient safety and outcomes but also contribute to a more efficient use of healthcare resources, ultimately benefiting both patients and healthcare organizations.

Several limitations should be acknowledged. First, with respect to study design, our review did not include randomized controlled trials. Although the included studies provide high-level evidence and high-quality appraisal, there is only one quasi-experimental study. Second, some studies lacked long-term follow-up, which precludes assessment of the sustained effects of the interventions. Third, the small number of included studies (n = 6), relative to the number of records initially identified, together with their methodological and clinical heterogeneity (different designs, settings, interventions, and outcome definitions), precluded statistical pooling and limited the generalizability of the findings. Fourth, restricting the search to studies published between 2021 and 2025 and to articles written in English or Portuguese may have excluded relevant earlier evidence and studies published in other languages, introducing a potential selection and language bias. Fifth, the eligible population encompassed all hospitalized adults aged 18 years or older across different clinical departments; because physical and cognitive function, comorbidity burden, and the risk and frequency of falls differ substantially between younger and older adults and between departments, this broad inclusion is a source of clinical heterogeneity. We did not apply a specific method to mitigate this variability beyond extracting and reporting the population and setting of each study; consequently, the pooled qualitative findings should be interpreted with this case-mix variability in mind. Finally, the definitions and denominators used to express fall outcomes (e.g., fall rate per 1000 patient-days versus the proportion of patients who fell, and the treatment of single versus recurrent falls) were not uniform across studies and were reported as published, which further limits direct comparability.

For future research, it is recommended to conduct randomized controlled trials. Additionally, it would be important to evaluate the cost-effectiveness of multifactorial nursing interventions to ensure the long-term sustainability of healthcare services, which is particularly relevant given the evolving demands of modern clinical practice. In terms of clinical implications, these findings suggest that nurses and multidisciplinary teams should prioritize standardized, multifactorial fall-prevention bundles that combine structured risk assessment, patient and staff education, and structured communication, embedded within institutional protocols and supported by continuous monitoring and audit. Technology-assisted monitoring (such as video monitoring during the night shift) may be considered as a complementary strategy for patients at high risk of falling, particularly where one-to-one observation is resource-intensive, provided that local cost and implementation factors are taken into account. Importantly, the consistency of effects appears to depend less on the specific intervention than on adherence and on tailoring the strategy to the clinical context, which reinforces the central role of nursing leadership and ongoing team training in translating these measures into sustained reductions in inpatient falls.

## 5. Conclusions

In summary, structured multifactorial, educational, and technology-based nursing interventions are generally associated with reductions in hospital fall rates. Although educational and communication-focused strategies show mixed outcomes, technology-assisted monitoring systems demonstrate some of the strongest effects on both fall reduction and resource utilization. Overall, the evidence supports the implementation of comprehensive, customized fall prevention guidelines that integrate fundamental nursing interventions with innovative technologies, thereby maximizing clinical effectiveness and enhancing patient safety.

## Figures and Tables

**Table 1:** Search strategy.

Database	Search Terms		Search Terms		Search Terms
CINAHL full-text	Inpatient * [b] OR Inpatient care [a] OR Inpatient services [b] OR Hospital setting [b] OR Hospital * [b]	AND	Accidental Falls [a] OR fall * [b] OR Falling [a] OR fall rate [b]	AND	Nurs * [b] OR Intervention [b] OR Nursing Care [a]
Medline	Inpatient * [b] OR Hospital setting [b]	AND	Accidental Falls [a] OR Fall * [b] OR Fall rate [b]	AND	Nurs * [b] OR Intervention [b] OR Prevention [b]
Scopus	Inpatient * [c] OR Hospital setting [c]	AND	Accidental Falls [c] OR Fall * [c] OR Fall rate [c]	AND	Nurs * [c] OR Intervention [c]

[a]—Exact Subject Heading; [b]—Title/Abstract; [c]—Title/Abstract and Keyword. The asterisk symbol * was used as a truncation operator, allowing different variations of the same lexical root to be retrieved.

**Table 2:** Risk of bias assessment.

Risk of Bias	Yost & Baur, 2021 [23]	Akhiwu et al., 2025 [24]	Kalafus et al., 2025 [25]	Rodríguez et al., 2023 [26]	Woltsche et al., 2022 [15]	Miles et al., 2025 [27]
Selection of participants	○	○	○	○	□	○
Confounding variables	□	○	○	□	○	○
Intervention measurement	○	○	○	○	○	○
Blinding of outcome assessment	□	X	○	□	X	X
Incomplete outcome data	○	□	○	○	X	□
Selective outcome reporting	○	○	○	○	○	○

Footnote: ○ = Low risk of bias; □ = High risk of bias; X = Unclear. Note: In Kalafus et al. (2025) [25], data collection was prospective from 1 February 2021 to 30 September 2022 and retrospective (historical reference period) from 1 June 2019 to 31 January 2021. As part of this period preceded the publication of the 2022 clinical practice guideline, a risk of bias related to the use of the previous protocol was considered when interpreting this study.

**Table 3:** Data extraction.

Author/ Year/ Country	Study Design	Population/ Context	Period and Duration	Nursing Interventions	Comparator	Measurements/ Instruments /Outcomes	Follow-Up (Period)	Results (Summary)	JBI Critical Appraisal (Total)
Yost & Baur, (2021) [23]—USA	Quality Improvement Study (Continuous Quality Improvement—CQI; Plan-Do-Study-Act—PDSA model) Pre/Post Audit; n = Not reported.	Adults admitted to a medical-surgical and stepdown neurology unit at an academic Comprehensive Stroke Center Hospital.	Pre-implementation data review: October 2018–June 2019; project implementation: July 2019; follow-up not precisely specified (outcomes monitored through 2-week PDSA cycles).	Implementation of a standardized fall risk assessment tool (Modified Morse Scale) and delirium screening (NuDESC) integrated into the electronic medical record (EMR); Nurse education and training (n = 60); Bundled interventions including bed and chair alarms, yellow armband, physiotherapy referral, mobility assistance, and nurse clinical decision-making; Patient and family educational materials (Fall TIPS™); Universal precautions pocket cards; Nurse leader rounding audits.	Pre-implementation period (usual care) vs. post-implementation period.	Fall risk scales: Modified Morse; Delirium: NuDESC (without QoL instrument).	Continuous monitoring throughout the project (exact dates not fully reported).	Reduction in fall rate: 5.177 → 4.79 per 1000 patient-days (≈−8%); increase in fall-free days (3 → 5).	9/11; 81.8%
Woltsche et al. (2022) [28]—Australia	Cohort study pre-post clinical evaluation n = 494 episodes of monitoring.	Adult inpatients identified as high falls risk; Focus on the night Shift Hospital.	N/A Implementation of portable video monitoring over 3 months (494 episodes); pre vs. post comparison over equivalent periods.	Overnight portable video monitoring (PVM) using commercial baby monitor equipment as an adjunct falls prevention strategy (from 8:00 p.m. to 7:30 a.m.). Nursing staff education and training via Zoom sessions; Nightly PVM registers completed by nurses; Nursing interventions triggered by PVM alerts (toileting, repositioning, pain management, hydration)—Enhanced supervision and security.	Pre-implementation period (usual care: hourly rounding and response to call bells) vs. post-implementation period.	PVM register (number of monitoring episodes, nursing interventions triggered, falls events); incident reporting; -Fall rate per 1000 bed days; -Total number of overnight falls; -Without QoL instrument; -Main outcome: falls.	Three months (duration of the intervention period); no long-term follow-up reported.	Reduction in nighttime falls and rate: 4.54 → 2.26 per 1000 bed-days (*p* = 0.003); nighttime falls 15 → 1.	9/11; 81.8%
Rodríguez et al. (2023) [26]—Spain	Observational prospective study; No randomization; groups defined by the adequacy of footwear brought by the patient.	Tertiary hospital—Hospital Clínico Universitario San Carlos. Units Geriatrics and Internal Medicine (n = 158) patients; Non-slip socks group n = 77 (48.73%); Adequate footwear group n = 81 (51.27%).	March 2022 End June 2022 Total duration 3 months.	Provision of non-slip socks to patients with inadequate footwear, assigned by trained nurses; Guidance and monitoring for safe footwear.	Adequate footwear versus non-slip socks (rubber sole, non-smooth, closed heel, no laces, Velcro fastened).	Downton Fall Risk Index; Patient characteristics, fall circumstances, consequence; Statistical analysis; Number of falls per group; Location, cause and consequences of falls; intrinsic and extrinsic risk factors.	Observation limited to the inpatient stay (up to 3 months)	137 patients did not fall: 77/77 from the non-slip socks group + 60/81 from the appropriate footwear group. There were 21 falls; all in the “appropriate footwear” group (*p* < 0.0001).	8/11 73.7%
Kalafus et al. (2025) USA [25]	Prospective data compared to a 20-month historical reference period (retrospective chart review). Single-site. No randomization or control group.	Gaylord Specialty Healthcare—137-bed Long-Term Acute Care Hospital.	Study period (prospective) 1 February 2021–30 September 2022 (20 months). Reference period (retrospective) 1 June 2019–31 January 2021 (20 months). Total observation 40 months. Mean CVM duration per patient 13.1 days.	Implementation of a continuous video monitoring (CVM) program using 12 AvaSure Guardian mobile telemonitor devices. Continuous video monitoring with dedicated technicians; activation criteria/protocols; coordination with nursing for rapid response; reduction of 1:1 surveillance.	Historical reference period (pre-CVM) 20 months of institutional data collected prior to program implementation (Jun 2019—Jan 2021), including inpatient fall reports and 1:1 sitter hours. No concurrent control group.	NA (outcome: falls) (without QoL instrument); Primary outcomes: Inpatient falls (total, per month, per 1000 patient-days); 1:1 sitter hours (total, per month, per 1000 patient-days, FTEs). Secondary outcomes: Adverse events avoided; cost analysis; patient satisfaction. Instruments: Institutional monthly fall reports; CVM system database logs; house nurse supervisor shift logs; patient satisfaction surveys (post-discharge, third-party vendor); chart review (age, sex, diagnosis, CVM status at time of fall). Statistical analysis and cost analysis.	40 months in total (20 + 20).	Reduction in average falls/month 17.2 → 12.9 (*p* = 0.02); reduction in surveillance hours 1:1 1428 → 140/month (*p* < 0.001).	9/11 81.8%
Akhiwu et al. (2025) [24]—USA	Evidence-based quality improvement (EBQI) project. Pre-post intervention design (before-after). n = 870 (pre); 879 (post-intervention).	Adult inpatients; Medical-surgical units Hospital.	N/A Pre-intervention (baseline)May, June, July (3 months) Intervention + post-interventionAugust, September, October (3 months). Total duration 6 months.	Fall prevention/control program with structured education for patients/family/staff and communication tools; reinforcement of documentation and adherence to preventive measures.	Pre-intervention period (same unit, 3 months prior).	N/A (outcome: percentage of falls) (without QoL instrument).	3 months (pre) + 3 months (post)	No difference in incidence: 0.9% (8/870) vs. 1.0% (9/879), *p* = 0.999; improved knowledge and adherence/documentation.	8/9 88.9%
Miles et al. (2025) [27]—Australia	Quasi-experimental mixed-methods study with embedded process evaluation. n = 684 (intervention) 476 (control).	Inpatients; Rehabilitation (rehabilitation inpatient care).	N/A Baseline (pre-intervention) May–August 2021 (4 months). Intervention delivery October 2021 (initial education + pre-survey). ImplementationNovember 2021 onwards. Follow-up education February 2022. Post-intervention survey April 2022. End of data collection July 2022. Total study period~14 months (May 2021–July 2022).	Teach-back communication intervention for inpatient falls reduction, delivered to all nursing, physiotherapy and occupational therapy staff on intervention wards	Usual care wards (Hospital B, 2 wards) versus interventions wards.	N/A (outcome: incidence of falls) (without QoL instrument).	Not reported (comparison by periods/units).	Falls: 2.2% (15/684) intervention vs. 6.1% (29/476) control (*p* = 0.211); significant reduction in subgroups (low risk; no cognitive impairment).	8/9 88.9%

## Data Availability

The original contributions presented in this study are included in the article. Further inquiries can be directed to the corresponding author.

## Supplementary Materials

The following supporting information can be downloaded at: https://www.mdpi.com/article/10.3390/nursrep16070232/s1, Table S1: PICO(S) framework and eligibility criteria; Table S2: Database-specific search strategies.

## Public Involvement Statement

There was no public involvement in any aspect of this research.

## Guidelines and Standards Statement

This manuscript was drafted using the JBI methodology for systematic reviews, and it was reported using the Preferred Reporting Items for Systematic Reviews and Meta-analyses guidelines.

## Abbreviations

The following abbreviations are used in this manuscript:

PRISMA	Preferred Reporting Items for Systematic Reviews and Meta-analyses
