# Supplementary material for: Nursing-Led Interventions for Preventing Falls in Hospitalized Patients: A Systematic Literature Review
Source: Nurs Rep. 2026 Jul 3;16(7):232. doi: 10.3390/nursrep16070232 (PMC13415614; doi:10.3390/nursrep16070232)
Supplement: Supplementary file 1 [file nursrep-16-00232-s001.zip › nursrep-4293410-supplementary.pdf]

Table S1. PICO(S) framework and eligibility criteria.

PICO(S)	Inclusion criteria	Exclusion criteria
**Population (P)**	Adults aged ≥ 18 years admitted to a hospital, regardless of the clinical department or specialty.	Outpatient populations;<br>Long-term care facilities without a hospital componente
**Intervention (I)**	Nursing interventions implemented in hospital settings aimed at preventing or reducing the incidence of falls, whether delivered as standalone measures or integrated within multidisciplinary programs.	Absence of an identifiable nursing intervention;<br>Ambiguous or insufficiently reported methodology.
**Comparison (C)**	Usual care, i.e., the standard fall-prevention practices in place at each setting prior to, or in the absence of, the evaluated nursing intervention	Studies without an identifiable comparison condition.
**Outcome (O)**	Primary: incidence of accidental falls among hospitalized adults<br>Secondary: changes in validated fall-risk assessment scores, adherence to fall-prevention protocols, and process indicators related to the implementation of nursing interventions.	Studies that did not provide sufficient fall-related outcome data.
**Study design (S)**	Experimental studies (randomized and non-randomized controlled trials);<br>Quasi-experimental studies;<br>Observational studies (cohort, case-control, and cross-sectional);<br>Published between January 2021 and December 2025; written in English or Portuguese.	Systematic reviews, meta-analyses, editorials, letters to the editor, case reports, opinion pieces, and narrative reviews;<br>Articles without full-text availability;

PICO(S): Population, Intervention, Comparison, Outcome, Study design.

Table S2. Database-specific search strategies.

CINAHL Complete (full text) — via EBSCO

Set	Concept	Search terms
#1	Population	(MH "Inpatient Care") OR TI (Inpatient* OR "Inpatient services" OR "Hospital setting" OR Hospital*) OR AB (Inpatient* OR "Inpatient services" OR "Hospital setting" OR Hospital*)
#2	Falls	(MH "Accidental Falls") OR (MH "Falling") OR TI (fall* OR "fall rate") OR AB (fall* OR "fall rate")
#3	Nursing intervention	(MH "Nursing Care") OR TI (Nurs* OR Intervention) OR AB (Nurs* OR Intervention)
**#4**	**Combination**	**#1 AND #2 AND #3**
	Limiters	Full text; publication date 2021-01-01 to 2025-12-31; language: English, Portuguese.

MEDLINE — via EBSCO

Set	Concept	Search terms
#1	Population	TI (Inpatient* OR "Hospital setting") OR AB (Inpatient* OR "Hospital setting")
#2	Falls	(MH "Accidental Falls") OR TI (Fall* OR "Fall rate") OR AB (Fall* OR "Fall rate")
#3	Nursing intervention	TI (Nurs* OR Intervention OR Prevention) OR AB (Nurs* OR Intervention OR Prevention)
**#4**	**Combination**	**#1 AND #2 AND #3**
	Limiters	Full text; publication date 2021-01-01 to 2025-12-31; language: English, Portuguese.

Scopus

Set	Concept	Search terms
#1	Population	TITLE-ABS-KEY (inpatient* OR "hospital setting")
#2	Falls	TITLE-ABS-KEY ("accidental falls" OR fall* OR "fall rate")
#3	Nursing intervention	TITLE-ABS-KEY (nurs* OR intervention)
**#4**	**Combination**	**#1 AND #2 AND #3**

Set	Concept	Search terms
	Limiters	AND PUBYEAR > 2020 AND PUBYEAR < 2026 AND (LIMIT-TO (LANGUAGE , "English") OR LIMIT-TO (LANGUAGE , "Portuguese"))

Field tags: *CINAHL / MEDLINE (EBSCO): MH = exact subject heading (CINAHL Headings / MeSH); TI = title; AB = abstract; Scopus: TITLE-ABS-KEY = title, abstract, and keywords; Truncation (*) retrieves word variants (e.g., nurs* = nurse, nurses, nursing)*
